# Bevacizumab Diminishes Inflammation in an Acute Endotoxin-Induced Uveitis Model

**DOI:** 10.3389/fphar.2018.00649

**Published:** 2018-06-19

**Authors:** Salvador Mérida, María Sancho-Tello, Inmaculada Almansa, Carmen Desco, Cristina Peris, Mari-Luz Moreno, Vincent M. Villar, Amparo Navea, Francisco Bosch-Morell

**Affiliations:** ^1^Departamento de Ciencias Biomédicas, Instituto de Ciencias Biomédicas, Universidad Cardenal Herrera-CEU, CEU Universities, Valencia, Spain; ^2^Department of Pathology, University of Valencia, Valencia, Spain; ^3^Department of Medical Ophtalmology, Fundación para el Fomento de la Investigación Sanitaria y Biomédica de la Comunitat Valenciana, Valencia, Spain; ^4^Department of Basic Sciences, Universidad Católica de Valencia San Vicente Mártir, Valencia, Spain

**Keywords:** bevacizumab, endotoxin-induced uveitis, inflammation, oxidative stress, chemokines, cytokines

## Abstract

**Introduction:** Uveitis is an eye disease characterized by inflammation of the uvea and an early and exhaustive diagnosis is essential for its treatment. The aim of our study is to assess the potential toxicity and anti-inflammatory efficacy of Bevacizumab in an experimental uveitis model by subcutaneously injecting lipopolysaccharide into Lewis rats and to clarify its mechanism.

**Material and Methods:** Blood–aqueous barrier integrity was assessed 24 h after endotoxin-induced uveitis (EIU) by analyzing two parameters: cell count and protein concentration in aqueous humors. Histopathology of all eye structures was also studied. Enzyme-linked immunosorbent analyses of the aqueous humor samples were performed in order to calculate the diverse chemokine and cytokine protein levels and oxidative stress-related markers were also evaluated.

**Results:** The aqueous humor’s cellular content significantly increased in the group treated with only Bevacizumab, but it had no effect on retina histopathological grading. Nevertheless, the inflammation noted in ocular structures when administering Bevacizumab with endotoxin was mostly prevented since aqueous humor cell content considerably lowered, and concomitantly with a sharp drop in uveal, vitreous, and retina histopathological grading. The values of the multi-faceted cytokine IL-2 also significantly decreased (*p* < 0.05 vs. endotoxin group), and the protective IL-6 and IL-10 cytokines values rose with related anti-oxidant system recovery (*p* < 0.05 vs. endotoxin group). Concurrently, some related M1 macrophage chemokines substantially increased, e.g., GRO/KC, a chemokine that also displays any kind of protective role.

**Conclusion:** All these results revealed that 24 h after being administered, Bevacizumab treatment in EIU significantly prevented inflammation in various eye structures and correct results in efficacy vs. toxicity balance were obtained.

## Introduction

Uveitis, an ophthalmological illness, has environmental and polygenic influences that bring about loss of vision and implicates many heterogeneous diseases, all characterized by intraocular inflammation, which commences in the uvea, and whose otherwise involved immune pathways have been precisely described (Trinh et al., 2008). Several causes are involved, e.g., infections and systemic autoimmune disorders.

Ocular inflammation involves mainly the uveal tract, but it can be extended to additional ocular structures, such as the vitreous or retina. The main invading immune cells are T-lymphocytes and monocyte-derived macrophages, which are located in growing lesions (Mérida et al., 2015). Both of these cell types release an array of soluble inflammatory mediators (chemokines and cytokines), which are vital for disease onset and disease progress (Sijssens et al., 2007). Some cytokines are protective or inflammatory, which depends on the time they take place. Apart from this complex paradigm, many other cytokines also have antithetical effects depending on the context in which they are released (Curnow and Murray, 2006).

Endotoxin-induced uveitis (EIU) is an acute uveitis procedure that can be caused by systemically injecting into rats a sublethal lipopolysaccharide (LPS) dose, a constituent of Gram-negative bacteria’s cell walls. Hence, it has not only been seen as a valuable model of human anterior uveitis and panuveitis (Rosenbaum et al., 1980; Lin et al., 2018), which is not autoimmune, but is also a useful model for procedures of acute ocular inflammatory driven by innate immune ways (Caspi, 2006). The application to humans of the results obtained in the EIU model is considered useful to study a part of the endogenous uveitis (the faculty of some bacteria and toxins to act on uveal tissues has been known since ancient times), some of which have been related to infections by gram negative bacteria (Misiuk-Hojło, 1998). EIU is characterized by iris vasodilatation and vascular alterations in the ciliary body, complemented by augmented vascular permeability and blood–aqueous barrier breakdown (Chen et al., 2009; Girol et al., 2013). In the eye’s anterior segment, an alteration of the blood barrier involves protein leakage into the anterior chamber and subsequent macrophage and neutrophil infiltration. Inflammation appears 4 h after injecting LPS, peaks after 24–48 h, and decays 96 h after inducing the disease (Rosenbaum et al., 1980; Trinh et al., 2008; Chen et al., 2009). There is plenty of experimental and clinical evidence that support the role of specific Gram-negative bacteria, or their LPS, in the pathogenesis of non-infectious immune-mediated uveitis (Chen et al., 2009).

Endotoxin-induced uveitis model mimics the pathologies in human acute uveitis (Rosenbaum et al., 1980; Lin et al., 2018). Therefore, in the EIU model, chemokines and cytokines are also important in the response. Thus, during animal model progress and modulation for not only panuveitis, but also for acute ocular inflammation (Rosenbaum et al., 1980), several chemokines and cytokines released by infiltrating cells, e.g., INFγ (interferon gamma), IL-1 (interleukin-1), TNFα (tumor necrosis factor alpha), IL-6 (interleukin-6), MCP-1 (monocyte chemoattractant protein-1), RANTES (Regulated on Activation, Normal T Cell Expressed and Secreted) and other inflammatory mediators, play an essential role (Trinh et al., 2008; Girol et al., 2013; Toguri et al., 2014). Type 1 T helper cells (Th1) activation in EIU appears predominant, but takes place with any kind of Type 2 T helper cells (Th2) contribution (Trinh et al., 2008). Thus, not only the release of cytokines/chemokines, but also LPS activation of mononuclear and neutrophil cells in eye tissues, induce the secretion of different substances, such as free radicals and proteolytic enzymes, which have been associated with oxidative stress (Zhang et al., 2007; Sande et al., 2014).

Bevacizumab is a full-length monoclonal antibody (149 kDa) that was designed and considered mainly to be an anti-angiogenic approach to treat a wide range of solid tumors (El-Kenawi and El-Remessy, 2013). After its approval by the US Food and Drug Administration (FDA), ophthalmologists administered Bevacizumab to treat a variety of neovascular diseases. In experimental models, vascular endothelial grow factor (VEGF) inhibition of choroidal neovascularization and diabetic retinopathy have obtained auspicious results (Saishin et al., 2003; Nicholson and Schachat, 2010). Bevacizumab use is now widespread to treat any ocular disease that implies neovascularization. Promising results have been obtained from intravitreal injections of anti-VEGF agents in other ocular diseases, such as retinopathy of prematurity (Nazari et al., 2010), neovascular glaucoma (Falavarjani et al., 2010), macular edema of diabetic patients (Wells et al., 2016) and intraocular tumors (Detorakis et al., 2012). Bevacizumab has been employed to treat and stabilize subsequent uveitis complications, e.g., uveitis macular edema (Mackensen et al., 2008), choroidal neovascularization (Fine et al., 2008), and retinal vasculitis (Mirshahi et al., 2009). Nevertheless, its use in primary defeat inflammation has never been established. And it has never been mechanistically assessed in an animal model. Poku et al. (2014) have recently evaluated if intravitreal Bevacizumab was safe as monotherapy in various eye diseases and reported low rates of severe adverse effects after intravitreal Bevacizumab treatment in comparison to other intravitreal therapies. However, in clinical practice undesired events and usual complications can occur when injecting intravitreal anti-VEGF agents, and they can be potentially severe (Falavarjani and Nguyen, 2013) such as endophthalmitis (Mccannel, 2011), intraocular inflammation (Tolentino, 2011), ocular hemorrhaging (Modarres et al., 2009), etc. Accordingly, we previously demonstrated that an intravitreal Bevacizumab injection *per se* generates transient and mild, yet immediate inflammation in rat eyes, which has not been related to oxidative stress in ocular tissues (Sancho-Tello et al., 2008). Such injections have also resulted in a several-fold increase in RANTES, MCP-1, and INFγ concentrations in the aqueous humors of rats treated with endotoxin (Johnsen-Soriano et al., 2010).

Our research was focused on the study of the possible effect and, for the first time, the mechanism of Bevacizumab. Bevacizumab is a monoclonal antibody that includes human framework regions, ∼93% human and 7% murine protein sequence, and the complementarity-determining regions of a murine antibody which binds to VEGF (Rini et al., 2004; Sharma et al., 2010). After several years questioning Bevacizumab-murine VEGF interaction (Liang et al., 2005; Manzano et al., 2006a; Bock et al., 2007; Gerber et al., 2007; Cheng et al., 2008; Lee et al., 2008; Yu et al., 2008; Shimomura et al., 2009) and its use in murine animal models, many recent works have confirmed its use in rats (Krempel et al., 2014; Lu et al., 2014). Indeed, recent experiments showed the affinity of fluorescent-labeled bevacizumab to recombinant rat VEGF_164_, but with a comparable lower affinity to the rat protein than to recombinant human VEGF (Meyer et al., 2016). The binding profile of Bevacizumab to human, mouse, and rat VEGF-A is similar when tested by direct enzyme-linked immunosorbent assay (ELISA) (Irani et al., 2016). Bevacizumab interacts with human VEGF-A at 21 residues (Muller et al., 1998). There is a single amino acid substitution in rat VEGF-A (Irani et al., 2016). This minor change at the binding site might elucidate why bevacizumab binding to rat VEGF-A is weaker. In fact, binding to rat VEGF-A is similar to human VEGF-A at five orders of magnitude higher antibody concentration (Irani et al., 2016).

## Materials and Methods

### Animals

Male Lewis rats, weighing 250–300 g, aging 10 weeks (Harlan Ibérica SL, Barcelona, Spain) were used in accordance with international EU (86/608/EEC), ARVO (Association for Research in Vision and Ophthalmology) and ARRIVE (Animal Research: Reporting of In Vivo Experiments) (Kilkenny et al., 2010; McGrath and Lilley, 2015) regulations on handling animals. The study was approved by the Ethics Committee of Animal Experiments at the Universidad CEU-Cardenal Herrera (Permit No. 315/2006). Animals were confined individually and remained in a 12 h/12 h light/dark cycle, with regulated temperature (20°C) and relative humidity (60%) and *ad libitum* access to food and water. Rats were anesthetized by intraperitoneally (i.p.) injection of ketamine (100 mg/kg body weight) and azepromazine (2.5 mg kg^-1^ body weight). Animals were divided randomly into five experimental groups. A drop of topical anesthetic (procaine + oxybuprocaine) was administered three times every 3 min previous to the intravitreal injection. In addition, one drop of antibiotic (Polymyxin B Sulfate, gramicidin, and neomycin sulfate) was administered before intravitreal injection and every 8 h afterward.

Endotoxin-induced uveitis (the E group, *n* = 14) was provoked by footpad injections of 200 μg LPS (100 μg per footpad) from *Salmonella typhimurium* (Sigma-Aldrich, St. Louis, MO, United States), diluted in 0.2 mL saline solution. The saline solution control animals received the same volume of saline solution that was given with LPS (the S group, *n* = 10). The biological effect of Bevacizumab in rat models has been attributed to the fact that binding to rat VEGF-A is similar to human VEGF-A at five orders of magnitude higher antibody concentration (Meyer et al., 2016). Vitreous volume in adult rat eye is about 50 μL (Sha and Kwong, 2006) and about 4 mL in human eye (Angi et al., 2012). Typical Bevacizumab human dose is 1.00–1.25 mg in adults. Therefore, 80 μg of Bevacizumab (Avastin, Genentech, United States) was the chosen dose, a dose which in vitreous humor rat eye is 5.1–6.4 orders of magnitude higher concentration than human typical dose. Immediately after LPS footpad injection, one group of rats was slowly injected intravitreally (i.v.) with Bevacizumab (group E+B, *n* = 12; 80 μg in 3 μL samples) diluted in saline solution. A second group of rats not injected with the previous LPS footpad was also administered with Bevacizumab (80 μg in 3 μL samples; group B, *n* = 10). Similarly, E group was injected with an intravitreal saline solution and a fourth group (S group) was injected with the same saline solution. Finally, a fifth group (C group, *n* = 10) did not receive any treatment. The treatments and quantity of rats used are shown in **Table 1**. Animals were monitored every 8 h until sacrifice.

**Table 1:** Summary of the treatment groups.

Footpad injection	Intravitreal injection	Treatment group	No. of animals
None	None	Control (C)	10
Saline	Saline	Saline (S)	10
Saline	Bevacizumab	Bevacizumab (B)	10
Endotoxin	Saline	Endotoxin (E)	14
Endotoxin	Bevacizumab	Endotoxin+Bevacizumab (E+B)	12

Animals were anesthetized with sodium pentobarbital injected intraperitoneally and sacrificed by cervical dislocation 24 h after Bevacizumab and/or LPS treatment. The aqueous humor was taken out and enucleation was carried out immediately. Eyes were collected in 10% buffered paraformaldehyde for histopathological evaluations or frozen at -80°C until being processed for biochemical analyses.

### Aqueous Humor Collection and Cell Counting

Immediately after sacrificing animals, aqueous humors were collected from both eyes by anterior chamber puncture with an insulin syringe, and around 20 μL per rat were obtained. Blood–aqueous barrier integrity was evaluated 24 h after inducing uveitis by assessing two parameters in aqueous humors: cell count and protein concentration. For cell count, an aqueous humor aliquot was diluted in an equal volume of trypan blue. Cells were counted by a hemocytometer under light microscopy. The number of viable/non-viable cells (the equivalent to 0.1 μL) was counted manually per field, whereas the number of cells per microliter was obtained by averaging the results of at least four fields from each sample. Having completed cell counts, aqueous humors were centrifuged for 5 min at 300 *g*. The cell-free supernatant was removed and frozen at -80°C until being used.

### Histopathological Evaluation

Eyes remained in buffered paraformaldehyde for 24–48 h and then they were paraffin-embedded in accordance with standard procedures. Sagittal sections (3-μm thick) were cut close to the optic nerve head, and He-stained (hematoxylin and eosin). The histopathology of all the ocular structures (cornea, lens, uvea, retina, choroid, sclera and anterior, posterior, and vitreous chambers) was studied, identifying extravasated inflammatory cells such as neutrophils and monocytes/macrophages. Two independent observers scored the histopathological inflammation signs using grades 0–3: no infiltrating cells “0,” mild “1,” moderate “2,” or severe “3” cell infiltration.

### Biochemical Analysis

Eyes were enucleated and lenses removed after assuming their elevated glutathione concentration. Lensless eyes were frozen at -80°C until treated for the biochemical analysis of oxidative stress markers: glutathione (GSH), malondialdehyde (MDA) concentrations, and glutathione peroxidase (GSH-Px) activity. Lensless eyes were homogenized in 0.2 M potassium buffer, pH 7.0 and were frozen at -20°C until used.

Protein concentration was quantified following the method of Lowry et al. (1951) with modifications (Peterson, 1977). The aqueous humor samples were submitted to ELISAs in order to measure the levels of diverse chemokine proteins (GRO/KC, MIP-2, and MIP-3α) and cytokine proteins (IL-1β, IL-2, IL-6, IL-10, TNFα, and INFγ). ELISAs (Searchlight Multiplex rat Assays; Pierce Biotechnology, Inc., Woburn, MS, United States) were performed according to the manufacturer’s guidelines.

Malondialdehyde was measured in eye homogenates by HPLC (Waters-LC Module I Plus, Waters Cromatografia SA, Spain) following Richard’s procedure (Richard et al., 1992) modified by Romero et al. (1998). HPLC was run in a Spheryc-5, ODS 5 μm, 250 mm × 4.6 mm column (Brownlee-Columns) at a 1 mL/min flow rate. Values were expressed as nmol/mg of protein.

Glutathione was similarly measured in eye homogenates by HPLC (Gilson International B.V. Spain) in accordance with the procedure of Reed et al. (1980). A 3-Spherisorb NH_2_ 5 μm, 250 mm × 4.6 mm column (Waters Cromatografia, SA, Spain) was used at a 1 mL/min flow rate. Values are expressed as nmol/mg of protein.

Glutathione peroxidase activity was evaluated spectrophotometrically by monitoring NADPH oxidation at 340 nm, in line with Lawrence et al. (1978). Values are expressed as nmol/min of NADPH oxidized per mg of protein.

### Statistical Analysis

Data are expressed as mean ± SE. Inter group comparisons were made by one-way analysis of variance (ANOVA) and false discovery rate-adjusted *P*-value [FDR] < 0.05. The ANOVA of the data acquired by the Brown-Forsythe test was run by the Tukey test as a *post hoc* test when variances of data were homogeneous (*p* < 0.05), or by a Dunnett T3 test when variances differed. Statistical differences were set at *p* ≤ 0.05. Statistical tests were carried out using GraphPad Prism version 7.04 for Windows (GraphPad Software, La Jolla, CA, United States) and SPSS statistical software package v24 (IBM, Corp. Released 2016. IBM SPSS Statistics for Windows, Version 24.0., Armonk, NY, United States: IBM, Corp.).

## Results

### Effect of Bevacizumab on Protein and Cellular Concentration in Aqueous Humors

**FIGURE 1:**
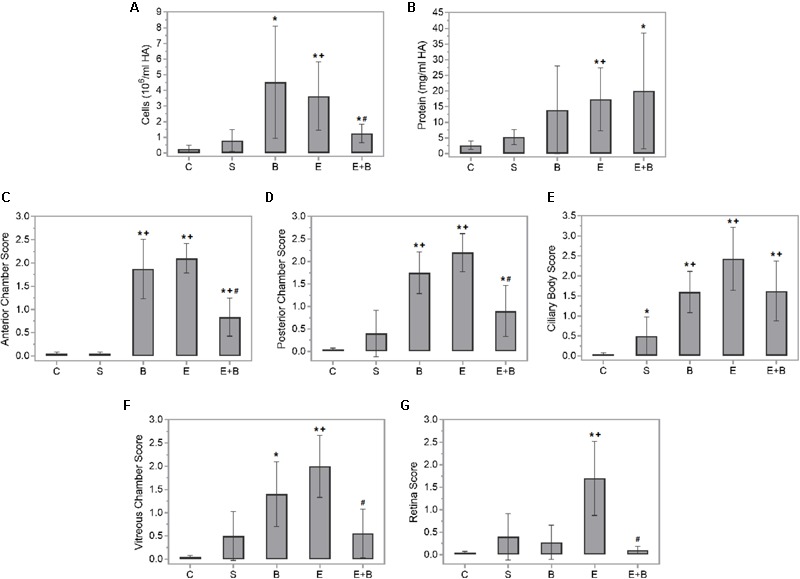
Effect of endotoxin and/or Bevacizumab on cellular content **(A)** and protein concentration **(B)** in the aqueous humors collected 24 h following treatment, and on the histopathological score of anterior chamber **(C)**, posterior chamber **(D)**, ciliary body **(E)**, vitreous chamber **(F)**, and retina **(G)** cellular infiltration. Each value is shown as mean ± SE. ^∗^*p* ≤ 0.05 vs. the Control group, ^+^*p* ≤ 0.05 vs. the Saline group and ^#^*p* ≤ 0.05 vs. the Endotoxin group.

Intense inflammation was observed in the rat anterior chamber of eyes 24 h after administering endotoxin, which indicates disrupted blood–barrier integrity. Hence, the inflammatory cellular content (*p* ≤ 0.05 vs. C and S groups; **Figure 1A**) and protein concentration (*p* ≤ 0.05 vs. C and S groups; **Figure 1B**) in the aqueous humors of the endotoxin-treated rats significantly increased. Moreover, statistically significant differences were observed in the inflammatory cellular content in aqueous humors between the data from the control group and the Bevacizumab groups (*p* ≤ 0.05 vs. C group; **Figure 1A**). A non-significant increasing tendency in the aqueous humor protein concentration tended to significantly increase in the Bevacizumab group (*p* > 0.05 vs. C and S groups; **Figure 1B**). Nevertheless, administering Bevacizumab with endotoxin moderately reduced the inflammation noted in the anterior chamber of eyes since a significant drop in cellular content was noted (*p* ≤ 0.05 vs. E group; **Figure 1A**), which was not found in the aqueous humor protein concentration (*p* > 0.05 vs. E group; **Figure 1B**).

### Histopathological Findings

**FIGURE 2:**
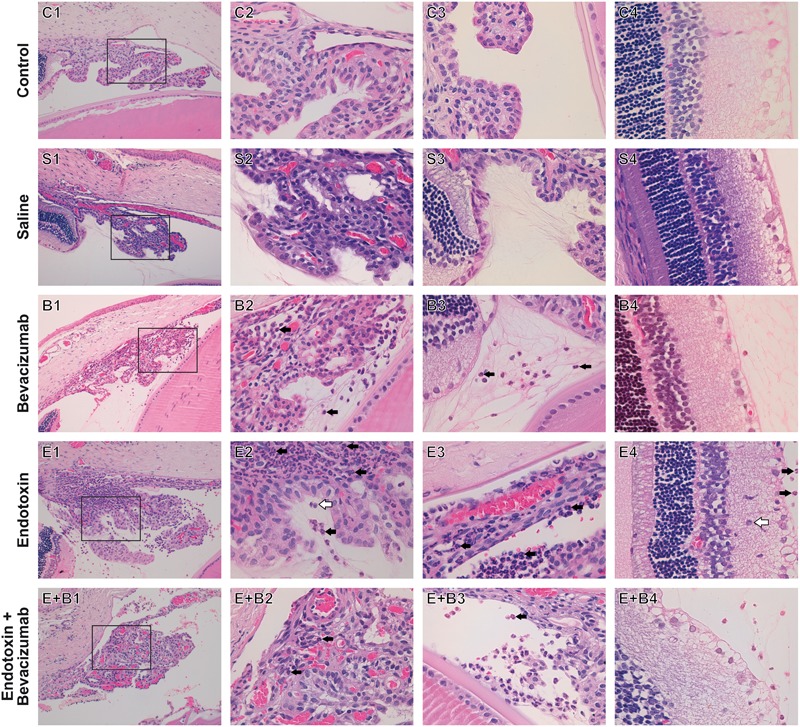
Histopathological study of ocular structures 24 h after Bevacizumab and/or endotoxin treatment, stained with HE. Picture number 1: Ciliary body, anterior and posterior chambers, 20x. Picture number 2: Inset of picture number 1, 63x. Picture number 3: Vitreous chamber, 63x. Picture number 4: Retina, 63x. Inflammatory cells were not observed in either the Control **(C)** or the Saline **(S)** group. Inflammatory cells were seen to infiltrate extravascular uveal tissue in Bevacizumab-treated eyes **(B)**, and reached all the studied structures, apart from the retina (ciliary body, anterior, posterior, and vitreous chambers). Many inflammatory cells neutrophils (black arrow) and monocytes/macrophages (white arrow), were found to infiltrate extravascular uveal tissue in the endotoxin-treated eyes **(E)** and reached all the structures under study (ciliary body, anterior, posterior and vitreous chambers, and retina). A significant reduction was noted when Bevacizumab was also injected (**E+B**, ciliary body, anterior, posterior and vitreous chambers, and retina).

Panuveitis was reached by endotoxin treatment and verified by the significant increase in the quantity of inflammatory cells in all the intraocular tissues studied (*p* ≤ 0.05 vs. C and S groups; **Figures 1C–G, 2C,S**) in histopathological sections (**Figures 2E1–E4**). The simultaneous administration of Bevacizumab mainly prevented endotoxin-induced uveitis (EIU) and a significant decrease was observed in the histopathological gradings of the anterior and posterior chambers (**Figures 1C,D**, *p* ≤ 0.05 vs. E group; **Figures 2E+B1,E+B2**). As mentioned above, endotoxin treatment led to a significant increase in the inflammatory cells in the ciliary body (*p* ≤ 0.05 vs. C and S groups, **Figures 1E, 2E1,E2**) and was more serious than in the anterior and posterior chambers (*p* ≤ 0.05 vs. C and S groups, **Figures 1C,D, 2E1,E2**). No significant decreasing tendency in endotoxin-induced ciliary body inflammation was noted when Bevacizumab was administered simultaneously (*p* > 0.05 vs. E group, **Figures 1E, 2E+B1,E+B2**). The number of inflammatory cells in the retina and vitreous chamber significantly grew after LPS treatment (*p* ≤ 0.05 vs. C and S groups, **Figures 1F,G, 2E3,E4**) and were found all over retinal layers. Administering Bevacizumab with endotoxin largely prevented inflammation in the retina and vitreous chamber (*p* ≤ 0.05 vs. E group, **Figures 1F,G, 2E+B3,E+B4**) and inflammatory cells were observed only in the innermost retinal layer (**Figure 2E+B4**). Interestingly, the inflammatory cell score in many intraocular structures sharply rose in the Bevacizumab group, which included the anterior and posterior chambers (*p* < 0.05 vs. C and S groups; **Figures 1C,D, 2B1,B2**), the ciliary body (*p* < 0.05 vs. C and S groups; **Figures 1E, 2B1,B2**) and the vitreous chamber (*p* < 0.05 vs. C group; **Figures 1F, 2B3**) but not the retina. The infiltration cell score for the retina was similar to that of the control and saline solution groups (**Figures 1G, 2B4**). Inflammatory cells did not appear in infiltrate lenses or the cornea in any study group.

### Bevacizumab Hinders Endotoxin-Induced Oxidative Stress

**FIGURE 3:**
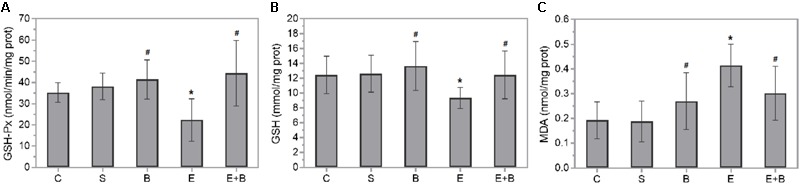
Effect of Bevacizumab on endotoxin-induced oxidative stress in rat eyes. Glutathione peroxidase activity **(A)**, glutathione **(B)**, and malondialdehyde **(C)** concentrations were measured in the eye homogenates of all the study groups. Each value is shown as mean ± SE. ^∗^*p* ≤ 0.05 vs. the Control and Saline groups, and ^#^*p* ≤ 0.05 vs. the Endotoxin group.

Diverse oxidative stress parameters were measured in rat eye homogenates 24 h after Bevacizumab and/or endotoxin treatment. Endotoxin significantly reduced GSH-Px activity and GSH levels (*p* ≤ 0.05 vs. C, S, and B groups; **Figures 3A,B**) which were completely recovered after simultaneous Bevacizumab administration (*p* ≤ 0.05 vs. E group, and *p* > 0.05 vs. C, S, and B groups). Likewise, endotoxin brought about a twofold significant increase in lipid peroxidation (*p* ≤ 0.05 vs. C, S, and B groups; **Figure 3C**), while simultaneous Bevacizumab administration inhibited lipid peroxidation because MDA concentrations reverted to control values (*p* ≤ 0.05 vs. E group, and *p* > 0.05 vs. C, S, and B groups).

### Bevacizumab Affects Chemokines and Cytokines in EIU

**FIGURE 4:**
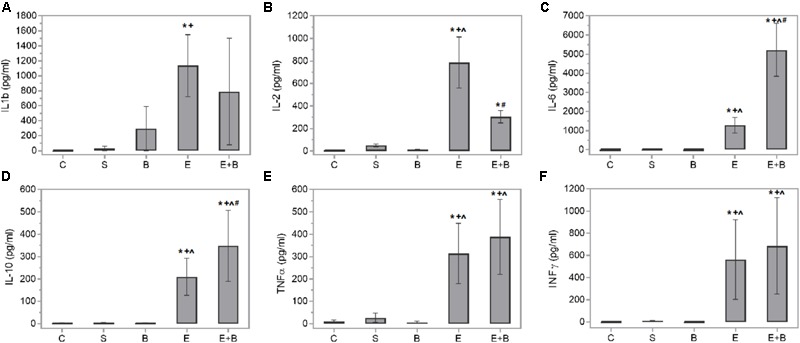
Effects of Bevacizumab on cytokine concentration (pg/mL) in the aqueous humors of all the study groups. IL-1b **(A)**, IL-2 **(B)**, IL-6 **(C)**, IL-10 **(D)**, TNFα **(E)**, and INFγ **(F)** concentrations were measured. Each value is shown as mean ± SE. ^∗^*p* ≤ 0.05 vs. the Control group, *ˆp* ≤ 0.05 vs. the Saline group, ^+^*p* ≤ 0.05 vs. the Bevacizumab group and ^#^*p* ≤ 0.05 vs. the Endotoxin group.

**FIGURE 5:**
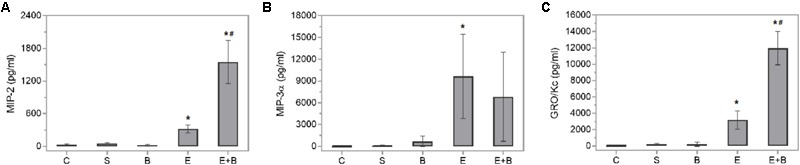
Effects of Bevacizumab on chemokine concentration (pg/mL) in the aqueous humors of all the study groups. MIP-2 **(A)**, MIP-3a **(B)**, and GRO/KC **(C)** concentrations were measured. Each value is shown as mean ± SE. ^∗^*p* ≤ 0.05 vs. the Control, Saline solution and Bevacizumab groups, and ^#^*p* ≤ 0.05 vs. the Endotoxin group.

Bevacizumab caused the protective IL-6 and IL-10 cytokines values and some macrophage-related chemokine values to rise after LPS treatment (**Figures 4C,D**). Systemic endotoxin injection significantly increased the aqueous humor levels of a wide range of inflammatory mediators, including all the measured cytokines (*p* ≤ 0.05 vs. C and S groups; **Figure 4**) and the control values increased between 30 and 100-fold. Simultaneous Bevacizumab and endotoxin administration led to a significant variation in IL-2 cytokine which, compared to the control values, increased 76-fold after administering endotoxin (**Figure 4B**) but decreased significantly when Bevacizumab and endotoxin were administered together (*p* ≤ 0.05 vs. E Group). Some monocyte-related cytokines (IL-6 and TNFα) increased when Bevacizumab was administered with endotoxin (**Figures 4C,E**). Cytokine IL-6 also rose considerably (*p* ≤ 0.05 vs. E Group; **Figure 4C**) under these conditions. IL-10 significantly increased after administering Bevacizumab and endotoxin together (*p* ≤ 0.05 vs. E Group; **Figure 4D**). Likewise, for cytokines, the LPS treatment led to significant increases in the concentration of all the chemokines measured in aqueous humor (*p* ≤ 0.05 vs. C, S, and B groups; **Figure 5**) and the control values rose between 12 and 500-fold. The effect that Bevacizumab had on endotoxin-stimulated chemokines was heterogeneous and caused no significant variations in MIP-3α compared to the E group (*p* > 0.05 vs. E Group; **Figure 5B**), while significant increases (*p* ≤ 0.05 vs. E Group; **Figures 5A,C**) were observed in two related macrophage chemokines: MIP-2 and GRO/KC.

## Discussion

Our results show the effects of Bevacizumab, a humanized anti-VEGF agent, in an animal model of non-autoimmune acute ocular inflammation. We found that Bevacizumab prevented endotoxin-induced inflammation in the anterior chamber of the eye because cellular content was reduced significantly and lowered histopathological grade obtained in anterior and posterior chambers (**Figures 1A–E, 2E+B1,E+B2**). Bevacizumab also prevented inflammation in the retina and vitreous chamber (**Figures 1F,G, 2E+B3,E+B4**). Bevacizumab inhibits VEGF from binding to its receptors (Flt-1 and KDR) on the endothelial cell surface and, as a result, neutralizes the biologic activity of VEGF. Thus, the significant reduction observed in both cell infiltration and histopathological grading of distinct ocular structures might be associated directly with this primary mechanism of action of Bevacizumab. VEGF displays vascular permeability via numerous mechanisms, including junctional remodeling, induction of fenestrae, and vesiculo-vacuolar organelles. Hence, VEGF like other inflammatory mediators (bradykinin, thrombin, histamine, etc.), disrupt the organization of integrin-extracellular matrix complexes and inter-endothelial junctions which open the junctional barrier. VEGF simultaneously activates several signaling pathways implicated in vascular permeability, including phospholipase C-dependent intracellular calcium release, eNOS signaling, cytoskeletal rearrangement, src kinase-mediated phosphorylation/internalization of junctional proteins and RhoGTPase activation. Therefore, minute intercellular gaps which are formed are allowing an unrestricted passage of plasma proteins, including liquid and albumin, to cross the endothelial barrier (Ferrara et al., 2003; Mehta and Malik, 2006). Recently, a study (Chalam et al., 2014) has associated treatment with Bevacizumab in age-related macular degeneration with IL-6 cytokine, and suggested that IL-6 could be a major marker of treatment response and resistance.

In the group treated only with Bevacizumab, it was noteworthy that the cellular content significantly increased in aqueous humor (**Figures 1A, 2B1–B3**). This phenomenon was noted in all the ocular structures that we studied (**Figures 1C–F, 2B1–B3**), except for the retina (**Figures 1G, 2B4**). In fact, the infiltration cell score obtained for the retina after administering Bevacizumab was similar to those for the saline solution and control groups. Other studies have similarly reported undesirable effects when higher intravitreal Bevacizumab doses were used, which resulted in ultrastructural alterations, transitory inflammation, and apoptosis in rabbit eyes (Manzano et al., 2006b; Avci et al., 2009). Our group has previously indicated that this effect disappeared within 1 week (Johnsen-Soriano et al., 2010). Angiogenesis is a necessary step to achieve wound healing. Some agents that impair blood vessel growth, e.g., Bevacizumab, could be expected to interfere with wound repair after an intravitreal injection. Following corneal epithelial injury in rabbits and rats, Bevacizumab can delay corneal epithelial wound healing, probably via the inhibited nerve growth factor expression (Kim et al., 2009, 2010).

All in all, and despite an early increase in inflammation, the fact that administering Bevacizumab (Bevacizumab group) did not alter retina histopathological grading is a very interesting finding. Bevacizumab with endotoxin (Endotoxin + Bevacizumab group) administration greatly prevented the inflammation observed in the retina at 24 h post-treatment (**Figures 1G, 2E+B4**). These findings indicate that Bevacizumab is effective treating acute uveitis. In any case, the retina in this acute model would be well-protected from any harmful effect that Bevacizumab could cause. No other animal model study has demonstrated retinal toxicity following intravitreal administration (Feiner et al., 2006; Shahar et al., 2006; Zayit-Soudry et al., 2011), despite the fact that Bevacizumab possibly interfere with initial postnatal retinal cell differentiation (Krempel et al., 2014) and with Müller glia activation (Gaddini et al., 2015).

We also measured relevant oxidative stress-related parameters in rat eye homogenates 24 h after Bevacizumab and endotoxin treatment (**Figure 3**). The E+B group showed a sharp rise in intracellular GSH levels vs. the E group. GSH is a usual thiol antioxidant peptide and co-substrate for detoxification enzymes, such as GSH-Px. Concurrently it increased GSH-Px activity, a selenium-containing enzyme which reacts with GSH molecules to control lipid peroxidation. Lipid peroxidation products, like MDA, might inhibit diverse enzymes, e.g., GSH-Px, in a concentration-dependent manner (Bosch-Morell et al., 1999, 2002). Hence, after Bevacizumab and endotoxin treatment, the high MDA concentration levels noted in the endotoxin-induced rat eyes reverted to the control values. We could attribute this antioxidant defensive outcome of Bevacizumab to some of the Bevacizumab effects observed in this study: first the marked drop in cellular infiltration; second some concomitant changes in the interleukin and chemokine concentrations.

There is abundant data that shows that chemokines and cytokines are relevant in uveitis development because they not only control the nature of immune responses (Foxman et al., 2002; Ilieva et al., 2004; Ooi et al., 2007), but also peak 24 h in ocular tissues or fluids in swollen eyes after an LPS injection (Steinke and Borish, 2006). Therefore, a high expression of different cytokines like TNFα, INFγ, IL-6, IL-1 and IL-2, has been simultaneously reported with a maximum EIU (de Vos et al., 1994; Xu et al., 2010; Mérida et al., 2013, 2014). Th1 activation also appears predominant in the EIU model, but with simultaneous Th2 participation (Trinh et al., 2008). Our data are consistent with the previous cited studies, such as Steinke and Borish (2006) and Trinh et al. (2008), because protein leakage and cellular content reached high levels 24 h after the LPS injection. We also observed various overexpressed chemokines and cytokines in our experimental uveitis model (**Figures 4, 5**). It is the case of IL-2, a cytokine that plays pivotal roles in the immune response. IL-2 is critical to the induction of the inflammatory immune response but also to the development and maintenance of T regulatory cells (Tregs). The Th1 lymphocytes-related cytokine IFNγ also rose in the endotoxin-treated animals vs. the control ones. Similarly, cytokines correlated to other inflammatory mediators, e.g., the Th2 lymphocytes ones (IL-10 and IL-6), or the macrophage ones (TNFα, IL-1β, and IL-6,) also showed high levels in this group.

Many of the cytokines that were measured herein showed the beneficial effect of Bevacizumab in EIU. IL-6 and IL-10, two cytokines that have anti-inflammatory properties, increased significantly when Bevacizumab and endotoxin were administered together. Numerous cell types release IL-10, including T- and B-cells (Th2 and Treg cells), monocytes, dendritic cells, macrophages, and mast cells. It seems to mitigate inflammatory responses by lowering the expression of major histocompatibility complex and co-stimulatory molecules upon the antigen presenting cells, and inhibits the release of pro-inflammatory cytokines (Trittibach et al., 2008; Saraiva and O’Garra, 2010). Only few studies have revealed that IL-6 is redox-regulated and may preserve organs exposed to oxidative stress. IL-6 may also induce neutrophils apoptosis and, therefore, contribute to neutrophil clearance (Scheller et al., 2011). When infiltration occurs at the inflammation site, as we can see at **Figures 2B,E,E+B** groups, neutrophils generate soluble IL-6 receptor (sIL6R) via proteolytic cleavage of the membrane-bound form (mIL6R). IL-6R proteolytic processing from invading neutrophils could subsequently promote IL-6 trans-signaling in resident tissue cells, which produces a switch from neutrophil recruitment to monocyte recruitment by largely increasing monocyte-attracting chemokines. Furthermore, some authors, such as Wruck et al. (2010), have proposed a role for IL-6 in oxidative stress defense. Nrf2, a redox-sensitive transcription factor, displays cell protection against electrophilic and oxidative stress. Nrf2 is also a great effective activator of antioxidant response element-dependent transcription. Wruck et al. (2010) revealed that Nrf2 is also a potent activator of IL-6 gene transcription *in vivo*.

IL-2 significantly decreased when Bevacizumab was administered with endotoxin. IL-2 is a pleiotropic cytokine that induces T-cell growth, enhances the cytolytic activity of natural killer cells, promotes Tregs differentiation, facilitates activation-induced cell death and inhibits IL-6-dependent signaling events, e.g., down-regulating IL-6 receptor expression (Banchereau et al., 2012). The effect that Bevacizumab had on endotoxin-stimulated chemokines varied. Several related M1 macrophage chemokines (GRO/KC and MIP-2) showed relevant increases (**Figure 5**). MIP-2 is a chemokine secreted by macrophages and monocytes that acts as a chemoattractant for polymorphonuclear white blood cells and hematopoietic stem cells. GRO/KC is expressed by epithelial cells, neutrophils, and macrophages. Some authors (Omari et al., 2008) have suggested that this chemokine could play any kind of protective role.

This has been the first time that Bevacizumab has been used in non-immune-mediated experimental uveitis with good results. It has been previously employed in humans to treat certain uveitis complications (macular cystic edema or neovascularization), but not as the primary treatment. Our results show the possibility of playing a role in such complex vision-threatening ocular diseases. Nonetheless, more studies are necessary to elucidate subjacent mechanisms of Bevacizumab treatment following vascular leakage in uveitis.

## Author Contributions

SM, MS-T, and FB-M conceived and designed the experiments. MS-T, IA, CD, and AN performed the experiments. SM and FB-M analyzed the data. SM, CD, CP, M-LM, VV, AN, and FB-M wrote the paper.

## Conflict of Interest Statement

The authors declare that the research was conducted in the absence of any commercial or financial relationships that could be construed as a potential conflict of interest. The reviewers SG and MP and handling Editor declared their shared affiliation.
